# Asymmetrical Induced Charge Electroosmotic Flow on a Herringbone Floating Electrode and Its Application in a Micromixer

**DOI:** 10.3390/mi9080391

**Published:** 2018-08-07

**Authors:** Qingming Hu, Jianhua Guo, Zhongliang Cao, Hongyuan Jiang

**Affiliations:** 1 School of Mechatronics Engineering, Qiqihar University, Wenhua Street 42, Qiqihar 161006, China; caoliang-8302@163.com; 2 School of Mechatronics Engineering, Harbin Institute of Technology, West Da-zhi Street 92, Harbin 150001, China; jhy_hit@sina.com

**Keywords:** asymmetrical induced charge electroosmotic, slip velocity, micromixer, herringbone floating electrode

## Abstract

Enhancing mixing is of significant importance in microfluidic devices characterized by laminar flows and low Reynolds numbers. An asymmetrical induced charge electroosmotic (ICEO) vortex pair generated on the herringbone floating electrode can disturb the interface of two-phase fluids and deliver the fluid transversely, which could be exploited to accomplish fluid mixing between two neighbouring fluids in a microscale system. Herein we present a micromixer based on an asymmetrical ICEO flow induced above the herringbone floating electrode array surface. We investigate the average transverse ICEO slip velocity on the Ridge/Vee/herringbone floating electrode and find that the microvortex generated on the herringbone electrode surface has good potential for mixing the miscible liquids in microfluidic systems. In addition, we explore the effect of applied frequencies and bulk conductivity on the slip velocity above the herringbone floating electrode surface. The high dependence of mixing performance on the floating electrode pair numbers is analysed simultaneously. Finally, we investigate systematically voltage intensity, applied frequencies, inlet fluid velocity and liquid conductivity on the mixing performance of the proposed device. The microfluidic micromixer put forward herein offers great opportunity for fluid mixing in the field of micro total analysis systems.

## 1. Introduction

The mixing of reactants has been found to be a very appealing and prominent operation in analytical preparation procedures, which has drawn extensive attention for its many practical applications, such as chemical reactions [1,2,3], protein crystallization [4,5,6], biological assays [7,8,9], biomedical diagnostics [10,11,12] and so on. However, since laminar flow is predominant and turbulence is usually not practically attainable in a microchannel or micro-reactor, mixing relies solely on molecular interdiffusion. Therefore, an efficient micromixer is essential for increasing the contact area and enhancing the mixing efficiency. Meanwhile, due to short mixing time associated with narrow diffusion length in laminar streams, the fast and efficient mixing of fluidic samples in microfluidic device is still challenging, especially at a low Reynolds number. To accomplish rapid homogeneous mixing, various interesting microfluidic mixing schemes have been developed to improve the mixing and residence time characteristics. Typically, the micromixer can be categorized as either passive or active [13,14,15]. A passive mixer always utilizes chaotic advection eddies induced by specific geometry microstructures to increase the interfacial contact area between adjacent liquids, including an in-plane mixer and an out-of-plane mixer. The in-plane passive microfluidic mixer characterized by splitting and reunification flows with specific configurations in the microchannel was employed to produce transverse dispersion, such as Tesla structures [16,17], micropillars [18], and staggered herringbones [19,20,21]. The complex three-dimensional architecture geometry structures within microchannel [22,23,24,25] was exploited to increase the contact area of the neighboring miscible fluids in the out-of-plane micromixer. Although the passive micromixer has the advantage of ease of integration into cascading microfluidic devices, the high pressure drop and the hydrodynamic dispersion potential of the sample deriving from spreading it out longitudinally always result in mechanical failure to a polydimethylsiloxane (PDMS) microchannel and loss of assay sensitivity, respectively. Alternatively, several active mixing approaches, including magnetic stirring [26,27], bubble-induced acoustic actuation [28,29,30], thermal field [31] and electrokinetically driven [32,33,34,35,36,37] mixing and so on, have been introduced into the development of a microfluidic mixer to disturb the interface of the fluids and enhance the mixing effect, namely as an active micromixer. Among these active schemes, the electrical field driven micromixers, which exploit the harmonic motion of thin electric double layers to induce interfacial disturbance when an electric field is applied on an electrolyte bounded by a dielectric, have attracted great attention due to their simple microelectrode structure, no mechanical moving parts and ease of integration. The commonly used electrokinetic phenomena for microscale fluid manipulation includes alternating current electroosmosis (ACEO) [38,39,40,41], dielectrphoresis (DEP) [42,43] and alternating current electrothermals (ACET) [44,45,46]. Arising from the interaction of an electric field with non-uniform permittivity and conductivity, the electrothermally induced micro-stirring was adopted to increase binding rates in heterogeneous assays and enhance the microfluidic immuno-sensors [46]. Undesired Joule heating may lead to electrolysis or bubble formation. Induced charge electroosmosis (ICEO), which refers to the electrokinetic phenomenon above the polarizable surface when the induced diffuse charge in a thin boundary layer acts with the tangential component of an applied electric field, provides new opportunities for fluid and particle manipulation. The ICEO micro-vortex above the flexible floating electrode has been utilized for particle focusing [47,48,49], fluid pumping [50,51] and cell trapping [52,53,54]. Additionally, many articles reported that this ICEO circulation flow around a highly polarizable conducting object circulation flow can disturb the interface of a two-phase flow and can be used to mixing fluidic samples [55,56,57]. Since the non-linear microvortex pair generated over the polarizable conductor-electrolyte surface in external electric field can disturb two-phase neighboring flow, many micromixers based on ICEO have been developed over the past few years. However, current ICEO-based micromixers mainly utilize a symmetrical microelectrode configuration to induce symmetrical eddies to mix the sample. Alternatively, it is expected that, by introducing a particular floating microelectrode configuration, asymmetrical microvortices can be obtained, which can be used to enhance species mixing.

In this work, we investigate the electrokinetic flow in a microchannel with different embedded floating microelectrode configuration deviating from the intermediate position of the driving electrode. An asymmetric micro eddies-based micromixer is proposed to achieve mixing enhancement with herringbone-conducting hurdles. We explore the parametric effect on the maximum slip velocity over the conducting electrode-electrolyte surface and the influence of floating electrode pair numbers on mixing performance. The dependences of the presented micromixer behavior on the applied voltage magnitude, electric field frequencies, inlet fluid velocity and liquid conductivities are also evaluated. The ICEO-based asymmetric microvortices scheme that is introduced offers a new possibility for rapid electrokinetic mixing with simple fabrication and easy operation in a modern microfluidic analysis system.

## 2. Theory and Methods

### 2.1. Micromixer Design

When immersed in an electrolyte, the electrical floating conductor attaching to the microchannel bottom wall becomes polarized with an electric field applied on the driving electrodes, causing the metal strip to become negatively charged near the anodic side while positively charged on the other side. The mobile charge carriers in the electrical double layer (EDL) interact with the tangential component of the imposed electric field and move to the oppositely neighboring charged electrode, inducing induce charge electroosmosis (ICEO) [35,48,58,59]. The ICEO enables a pair of symmetrical microvortices above the electrode surface, yielding liquid circulation motion.

**Figure 1:**
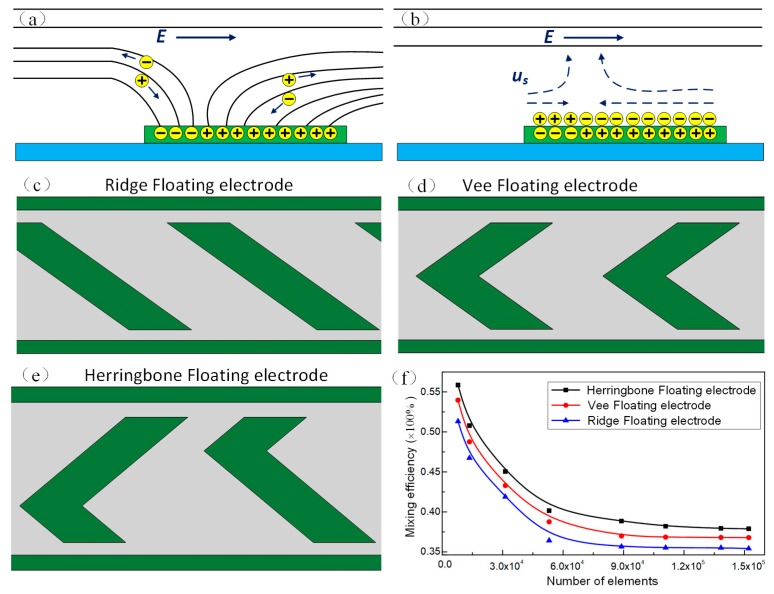
Schematic of induced charge electroosmotic (ICEO) flow on the polarizable metal strip and the ICEO slip velocity on the floating electrode with some deviation from the middle of driving electrodes. (**a**) The formation of the electric double layer on the floating electrode under the imposed external electrical field; (**b**) the asymmetrical ICEO microvortices above the polarizable floating electrode surface; (**c**–**e**) the Ridge/Vee/Herringbone type floating electrode microstructure; (**f**) the mesh independency test and the mixing efficiency at the outlet for the three types of metal strip with single electrode pair under the conditions of *A* = 5 V and *f* = 400 Hz.

Before designing the micromixer, we first conduct a simulation analysis to investigate the flow behavior and explore the slip velocity on the polarizable conductive object deviated from the middle of two driving electrodes. As shown in Figure 1a, owing to electromigration, the charge carriers in the electrolyte follows the initial normal electric field component to the metal strip surface when the driving electrodes are energized [60]. When the EDL is fully charged, an induced double layer formed at the electrolyte/floating electrode surface. The ionic charge in the diffusion layer travel under the action of tangential component of electric field, resulting in an asymmetrical microstream (Figure 1b), which can be utilized for fluidic mixing under certain conditions. To investigate the effect of micro metal strip structure on the slip velocity, we exploit three types of floating electrodes, namely Ridge, Vee and herringbone floating electrodes, as illustrated in Figure 1c–e. The ICEO slip velocity on polarizable surfaces under AC forcing can be exploited to disturb the fluid interface between two neighboring streams. Generally, the faster slip velocity at the electrode can produce stronger flow disturbance, resulting in better mixing effect [55]. To make sure the credibility of the simulation results, a grid-independence test is carried out to determine the suitable number of elements [61]. Comparing the tetrahedral meshes with the hexahedral meshes, since the hexahedral meshes have better computational accuracy and faster convergence, the computational domain was completely covered by the hexahedral meshes. Eight different structured hexahedral grid models with an element number ranging from 7.85 × 10^3^ to 1.52 × 10^5^ are tested. The mixing efficiency is simultaneously calculated under different element numbers. As shown in Figure 1f, the mixing efficiency varies slightly with the increasing element numbers beyond 1.10 × 10^5^. Therefore, a grid system with 1.10 × 10^5^ hexahedral elements is selected as the suitable grid system in terms of the accuracy and efficiency of simulation. We can also see from Figure 1f that the mixing efficiency for the herringbone floating electrode is better than the Ridge and Vee type floating electrode when the inlet flow velocity and the bulk conductivity are set to be 500 μm/s and 0.001 S/m under the conditions of *A* = 5 V, *f* = 400 Hz. It can be ascribed to the higher transverse slip velocity generated above the herringbone floating electrode under certain conditions. Therefore, we choose the herringbone floating electrode microstructure for further investigation.

**Figure 2:**
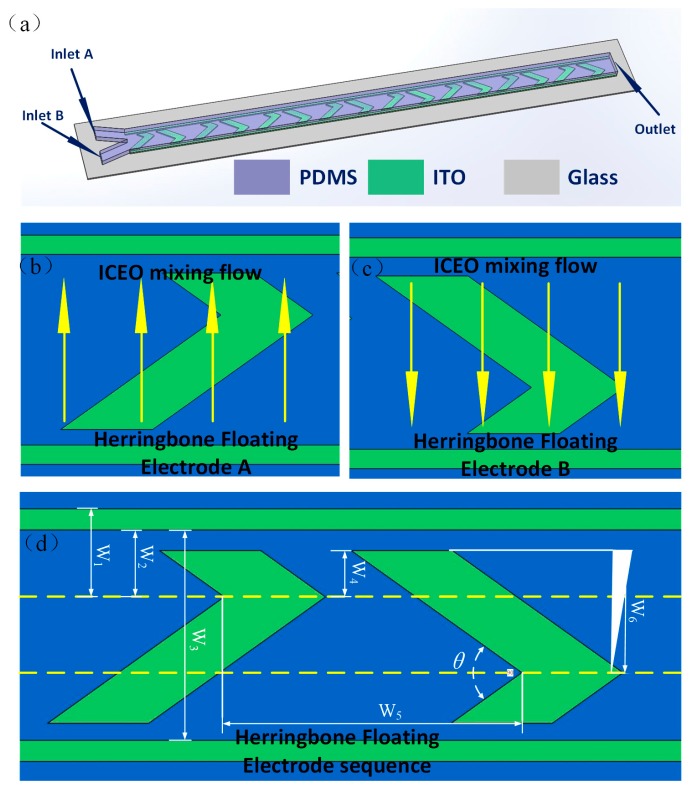
Geometry of proposed micromixer based ICEO. (**a**) 3D schematic diagram of the device with herringbone floating electrode sequence; (**b**,**c**) the fluidic sample motion on the left and right herringbone floating electrode surface under the asymmetrical ICEO vortex; (**d**) the specific dimensions of the microfluidic mixer device.

As depicted in Figure 2a, we explore a micromixer utilizing the asymmetric eddies above the ideally polarizable herringbone ITO (Indium Tin Oxide)-conducting microstructures in a straight polydimethylsiloxane (PDMS)/glass microchannel with a rectangular cross section of height and width 80 μm and 200 μm, respectively. The original fluidic sample can be injected into the Y-shaped entrance from inlet A and B. When an external AC signal is employed on the driving electrodes, which is perpendicular to the microchannel length direction, a pair of asymmetrical micorvortices are formed due to the asymmetrical configuration of herringbone floating electrode. Left and right herringbone floating electrode are defined in Figure 2b,c, respectively. The left herringbone floating electrode structure layouts will generate asymmetrical profile of micro vortex pairs, resulting in adjacent fluids interface being disturbed and the two-phase fluid samples being transported from right to left sidewall of the microchannel (Figure 2b). Similarly, the asymmetrical eddy pairs induced by the right herringbone metal strip will also break the samples’ interface and deliver them from left to right sidewalls (Figure 2c). Additionally, the feature sizes of the staggered herringbone floating electrode sequence configuration are specified as Figure 2d and the specific dimensions adopted in this work are given in Table 1.

### 2.2. Mathematical Model

Given electrochemical polarization theory, we take the charged double layers as a linear equivalent circuit and give a mathematical depiction of the micromixer-based asymmetrical ICEO microvortexes on the floating electrode. It is assumed that the charge carrier distribution in the bulk fluid is homogenous, the electrostatic potential satisfies Laplace’s equation [62]:

(1) $$\nabla^{2}\phi_{f}=0,\;\sigma_{f}\nabla \phi_{f}\cdot n=\frac{dq}{dt}$$

where $\phi_{f}$ is the electrostatic potential and *q* refers to the surface screening charge density of the induced double layer, *σ_f_* denotes the bulk conductivity, and ***n*** represents the normal vector in the interface of the bulk and floating electrode, pointing from the electrode into the bulk electrolyte.

The ohmic current from the bulk fluid resistance charges the induce double layer at the surface of the floating electrode can be regarded as a capacitor skin in the asymptotic limit [34]. In addition, the electric current flux flow at the interface of the bulk and floating electrode is continuous. Thus, this interface needs to be provided with the following physical constraint [63]:

(2) $$\left(\sigma_{f}+j\omega \varepsilon_{f}\right)n\nabla {\tilde{\phi }}_{f}=j\omega \frac{C_{D}}{1+\delta }\left({\tilde{\phi }}_{f}-{\tilde{\phi }}_{b}\right)\;$$

where *ε_f_* and *w* are the bulk permittivity and the angular frequency of the applied electric field, *δ* = *C_D_*/*C_S_* signifies the surface physical capacitance ratio of the diffuse layer *C_D_* = *ε_f_/λ_D_* to the stern layer *C_S_*, and *ϕ_b_* is the transient potential values at the metal surface. Here we introduced the complex phasor amplitude of each electrical field variable as denoted by a tilde for analytical convenience [56], e.g., ϕ(t)=Acos(ωt+θ)=Re(Aejθejωt)=Re(ϕ˜ejωt), where, Re( ) is the real part of ( ). At low frequency, there is no displacement current running through the double-layer capacitor skin. For the impenetrability of the ionic species, the sum of diffusion current and ohmic current in the bulk is equal to another one in the diffusion double layer.

When the phasor amplitude is applied on the left and right driving electrode, we can derive the electric potential distribution on the floating electrode without considering the double-layer polarization around the driving electrodes [47]:

(3) $${\tilde{\phi }}_{f}=A\;(\mathrm{on}\;\mathrm{the}\;\mathrm{left}\;\mathrm{driving}\;\mathrm{electrode}),\;{\tilde{\phi }}_{f}=0\;(\mathrm{on}\;\mathrm{the}\;\mathrm{right}\;\mathrm{driving}\;\mathrm{electrode})$$

As the electric double layer is completely charged, no normal electric field penetrate the floating electrode and the normal current flux disappears:

(4) $$n\cdot \nabla {\tilde{\phi }}_{f}=0\;$$

Based on the generation of the Helmholtz–Smoluchowski formula, the counterion on the polarizable floating electrode surface under the action of tangential electric field will travel with the transient ICEO velocity, which can be derived as,

(5) uslip=−εfηRe((ϕ˜b−ϕ˜f)1+δejωt)Re(E˜tejωt) 

where, *η* and ***E****_t_* denote the dynamic viscosity of aqueous media and the tangential field component on the floating electrode surface. In addition, the time-average non-linear electroosmotic slip velocity in AC oscillation can be expressed by:

(6) 〈uslip〉=−εf2ηRe((ϕ˜b−ϕ˜f)1+δE˜t*) 

As we know the above electroosmotic slip velocity on the floating electrode, we can employ it as a boundary condition and the full stokes equation in the bulk liquid, and the bulk fluid flow outside the EDL in the microchannel driven by the interfacial non-linear electrokinetic slip can be derived by the incompressible Stokes equations [64]:

(7) $$-\nabla p+\eta \nabla^{2}u=0\;$$

(8) ∇u=0 

where *p* and ***u*** are hydraulic pressure and fluid velocity vector, respectively.

The motion of fluidic sample in the microchannel are governed by many factors, including Brownian motion, Poiseuille flow and ICEO microscream. Therefore, we should take the convection–diffusion equation to describe the concentration of sample in the microchannel [65]:

(9) ∇(uc−D∇c)=0 

where *c* is the concentration of fluidic sample and *D* denotes the mass diffusivity of the target analyte.

### 2.3. Numerical Simulation

We adopt a commercial software package (V5.2, COMSOL AB., Stockholm, Sweden) to conduct numerical simulation so as to study the flow pattern of the microstream induced by the ICEO vortex on the herringbone floating electrode sequence surface and the mixing performance of the micromixer. The calculation process can be expressed as follows:

Firstly, in the Electric Currents module, we employed the electrostatic potential phasor *ϕ* = A and *ϕ* = 0 on the left and right driving electrode, respectively. The distribution impedances are employed in the driving electrode $n\cdot J=\frac{1}{d_{s}}\left(\sigma +j\omega \varepsilon_{0}\varepsilon_{r}\right)\left(\tilde{V}-{\tilde{V}}_{\mathrm{ref}}\right)$. The Laplace equation is prescribed in the bulk fluid and the electric field can be obtained from the potential as E=∇ϕ. For the impenetrability of the electric field on the surface of the floating electrode, the condition is employed on the floating electrode. The remaining boundary is set to electrical insulation with the condition n⋅J=0.Under these conditions, we calculate the electrostatic potential in the fluid domain and PDMS channel sidewall.

Secondly, in the Creeping Flow module, the full Stokes equations play the governing equation role and are then employed in the fluid domain. The pressure-driven flow with parabolic profile and surface averaged flow velocity *u*_0_ is prescribed to the both inlets for forward transport incoming fluid flow. Meanwhile, the pressure for the outlet flow is zero. Based on the above calculated electric field distribution, the slip velocity *u*_slip_ can be obtained and employed on the herringbone floating electrode sequence. The microchannel sidewalls surface expect the electrodes to be imposed as non-slip.

Finally, in the Transport of Dilute Species module, the convection–diffusion equation is employed in the fluid domain to calculate the concentration distribution of the fluid sample.The Inlet A and B is set to inflow 1 with *c* = 1 and inflow 2 with *c* = 0, the outlet is set to outflow with $-n\cdot D_{i}\nabla_{ci}=0$. In this way, we can estimate the mixing performance at the outlet plane. The remaining sidewalls are set to no flux $-n\cdot N_{i}=0$.

The following basic parameters are adopted in the numerical simulation: the conductivity of buffer solution is set to be *σ_f_* = 10 μS/cm, the dynamic viscosity is 0.001 Pa·s, and the diffusivity of the fluid sample is 2 × 10^−9^ m^2^/s.

### 2.4. Evaluation of the Mixing Efficiency

In order to evaluate mixing efficiency, here we defined a parameter *γ* to describe the mixing performance of the micromixer-based floating electrode in terms of the sample concentration at the outlet:

(10) $$\gamma =\left(1-\frac{\iint_{S}\left|c-0.5\left[{\mathrm{mol}/m}^{3}\right]\right|dS}{\iint_{S}\left|0.5\left[{\mathrm{mol}/m}^{3}\right]\right|dS}\right)\times 100\%\;$$

where *c* refers to the molar concentration of sample at the outlet plane. It describes the homogeneity of the mixed sample and denotes *γ* = 0% and *c* = 0 or *c* = 1 when the sample is not processed at any position, signifying that no mixing occurs. By contrast, this index can reach up to 100% and the *γ* = 100% at the exit if the sample is mixing completely.

## 3. Results and Discussion

### 3.1. The Microstream Driven by Induced Charge Electroosmotics (ICEO) in the Channel

**Figure 3:**
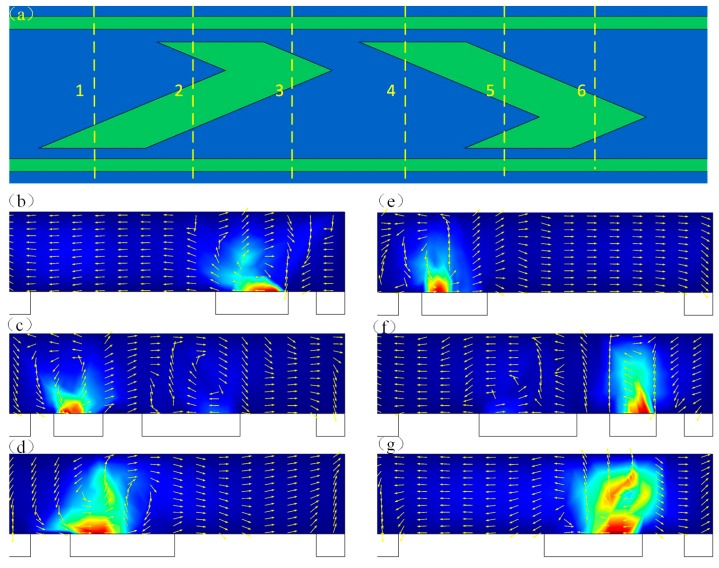
The fluid microstream at different cross section. (**a**) the definition of cross section 1–6; (**b**–**d**) the fluid motions on the left herringbone floating electrode at the cross section 1–3; (**e**–**g**) the fluid flow on the right herringbone floating electrode at the cross section 4–6.

An investigation on the fluid flow patterns induced by the ICEO asymmetrical vortex can provide a theoretical basis for developing a rapid and excellent micromixer. For this, we firstly concentrate the microstream on different cross sections of the microchannel under static conditions with no inlet flow (Figure 3a). With the driving electrodes energized by an AC voltage signal of suitable field frequencies, asymmetrical vortex pairs on the left herringbone floating electrode come into being after a typical double-layer relaxation time. From cross section 1, we know that the two fluid flows will be mixed by chaotic convection on the floating electrode surface and the mixed sample is transported to the opposite side wall (Figure 3b). At the cross section 2, the vortex close to the right driving electrode can mix and deliver the sample to the right side wall, while the ICEO vortex close to the left deriving electrode can enhance the mixing and fluid transportation performance (Figure 3c). At the cross section 3, the fluidic sample near the left driving electrode is mixed by the vortex pair and delivered to the right (Figure 3d). Interestingly, in the sections 4–6 the fluid motions are diametrically opposite to the cross sections 1–3 (Figure 3e–g), which will improve the mixing performance evidently. It can also be noted that the maximum electroosmotic slip velocity occurs in the vicinity of the floating electrode edge. In this way, fluidic sample can be transferred from one side to another side under the action of the ICEO electroconvective vortex and the interface is be disturbed repeatedly on the left and right herringbone floating electrodes surface, which can be exploited to accomplish excellent mixing.

### 3.2. The Electroosmotic Flow Velocity near the Floating Electrode

**Figure 4:**
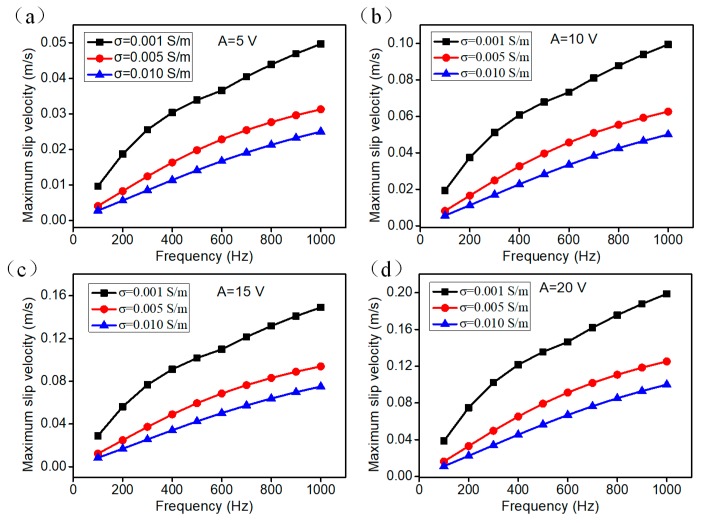
The frequency dependency of maximum slip velocity on the herringbone floating electrode at different voltage input and liquid conductivity. (**a**–**d**) the maximum slip velocity vs frequency when the voltage intensities are 5 V, 10 V, 15 V and 20 V at the liquid conductivity *σ* = 0.001 S/m, 0.005 S/m, 0.010 S/m.

The electroosmotic flow velocity above the floating electrode surface plays an important role in enhancing the fluid-mixing efficiency. We quantificationally studied the frequency dependency of the electroosmotic slip velocity on the floating electrode under different bulk conductivities (Figure 4). The frequency dependency of the maximum slip velocity on the herringbone floating electrode under different buffer solution conductivities at *A* = 5 V is shown in Figure 4a. Evidently, the lower electrical conductivity (*σ* = 0.001 S/m) will induce larger slip velocity, and the higher ion concentration (*σ* = 0.01 S/m) will weaken the effect of the ICEO flow. The reason for that is the Debye screening thickness reduces with the increasing ion concentration, which results in the weakness of the ICEO electroconvective flow field. It is noticeable that the increasing rate of maximum slip velocity decay is obviously near the inverse RC time constant, *f* = 300 Hz. One reasonable explanation is that the IDL (Induced Double Layer) have enough time to be fully charged at the frequency lower than the ionic charge relaxation frequency as the ICEO slip velocity is proportional to the quadratic of electric field intensity applied (Equation (6)). When the voltage intensity increased to be 10 V, the maximum slip velocity is twice as large as it is at *A* = 5 V. Similarly, the lower ion concentration will induce larger slip velocity. When the voltage input is increased to 15 V, 20 V, the slip velocity is enlarged obviously (Figure 4c,d), engendering stronger ICEO vortex streaming for efficient mixing.

### 3.3. Mixing Performance of the Device with Different Number of Herringbone Floating Electrode Pairs

**Figure 5:**
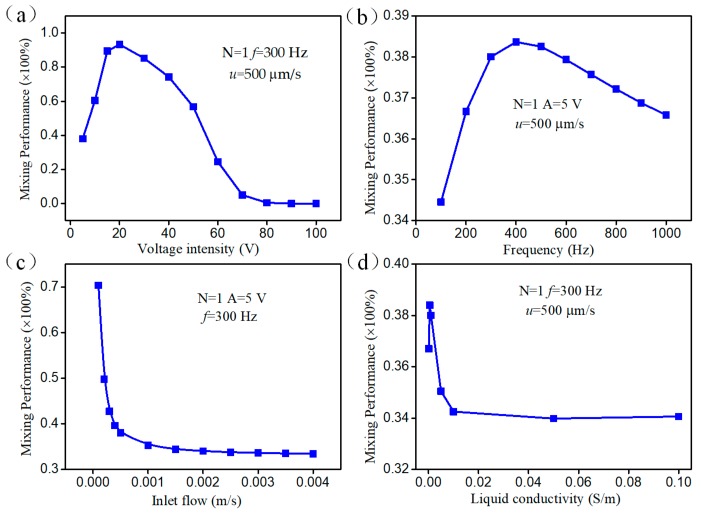
The mixing performance of the micromixer with a pair of herringbone floating electrodes. (**a**) The effect of voltage intensity on mixing performance at *f* = 300 Hz, *u* = 500 μm/s; (**b**) the frequency dependency of mixing performance at *A* = 5 V, *u* = 500 μm/s; (**c**) the relationship between the mixing performance and inlet flow at *A* = 5 V, *f* = 300 Hz; (**d**) the mixing performance under different liquid conductivity at *A* = 5 V, *f* = 300 Hz and *u* = 500 μm/s.

In this section, we make an attempt to explore the parametric effect on mixing behavior for engendering better performance by conducting simulation in the device with a pair of herringbone floating electrodes. Figure 5a) illustrates the relationship between the mixing performance and applied voltage amplitude at a fixed field frequency of *f* = 300 Hz while the inlet flow velocity is maintained at 500 μm/s. Owing to the complex configuration of the herringbone floating electrode, the ICEO slip flow generated on the entire electrode surface can be divided into the transverse and radial slip velocities. As the ICEO slip velocity at the solid/electrolyte interface has quadratic dependence on the electric field (Equation (6)), the ICEO transverse vortex flow can be utilized to enhance the convective flow and interface disturbance when the axial inlet flow velocity is constant. We can also see from the calculation results that the mixing performance increases with increasing amplitude ranging from 5 V to 20 V, which is in accordance with the theoretical prediction. However, mixing efficiency reaches the peak value at 20 V and diminishes with increasing voltage amplitude. In addition, the mixing efficiency becomes saturated to some extent at higher electric field strength above 80 V, exhibiting a poor mixing performance with increasing voltage amplitude. That could be ascribed to the influence of the radial slip velocity component, which could hinder the inlet fluids flowing into the microchannel and weaken the mixing efficiency when the axial ICEO flow is opposite to inflow direction and resembling the inlet flow velocity if the external electrical field exceeds a certain threshold. Field frequency is another important factor influencing ICEO convective slip velocity. The effect of varying field frequencies on the mixing performance is given in Figure 5b. At around 400 Hz, the mixing performance reaches a peak, whereas the maximum value is only 0.383. At high frequencies above 1000 Hz, the transverse weakened ICEO convective flow is not strong enough to transport the fluid quickly, which is not beneficial to fluid mixing. On the other side, mixing performance decays sharply with the increasing inlet fluid flow (Figure 5c). The reason behind this phenomenon is that the fluid samples have no sufficient time to interact with one another despite the disturbance of ICEO asymmetrical vortex pairs. It can be seen from the Figure 5d that the device has the greatest mixing performance when the electrical conductivity of the solution is 0.001 S/m. With the liquid conductivity increasing, the effect of ICEO is suppressed, resulting in the mixing performance beginning to decay and the mixing efficiency stabilizes at around 0.34.

**Figure 6:**
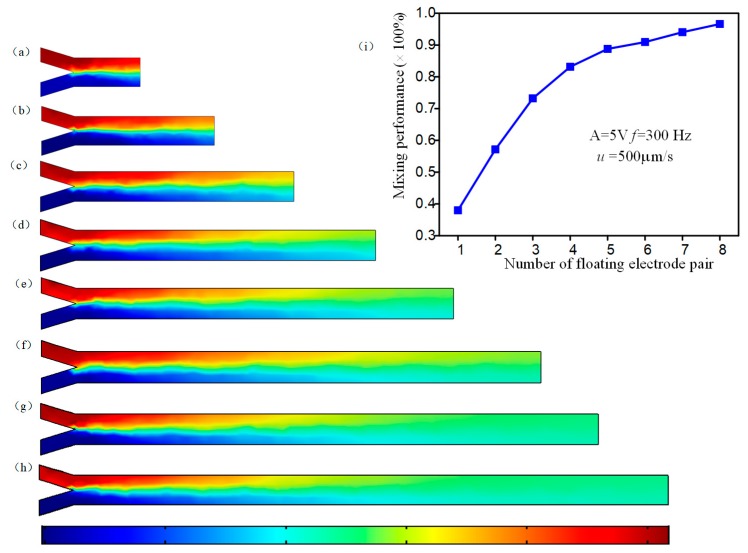
Mixing performance of the device with different number of herringbone floating electrode pairs. (**a**–**h**) The simulation results of the micromixer with different amount of Herringbone floating electrode pair; (**a**) a pair; (**b**) two pairs; (**c**) three pairs; (**d**) four pairs; (**e**) five pairs; (**f**) six pairs; (**g**) seven pairs; (**h**) eight pairs; (**i**) the curve illustrating the mixing performance and the number of herringbone floating electrode pair.

To accomplish efficient mixing, we also explore the mixing performance of the micromixer with different numbers of herringbone floating electrode pair sequentially configured on the microchannel bottom while the AC voltage amplitude is fixed at *A* = 5 V and the field frequency *f* = 300 Hz and inlet flow *u* = 500 μm/s. As exhibited in Figure 6, we can find that more herringbone floating electrode pairs can increase the opportunities of ICEO asymmetrical eddies to transfer the fluidic sample, enlarging the mixing region and improving sharply the mixing performance (Figure 6a–h). The quantitative relationship of mixing performance and the amount of herringbone floating electrode pairs is given in the Figure 6i. When the micromixer has five pairs of floating electrodes, the mixing performance can exceed 0.9. With the number of herringbone floating electrodes increases, the mixing percentage tends towards a steady state of around 0.96.

### 3.4. Parametric Effect on Mixing Performance

To understand the influence of factors on the performance of the micromixer, we study the chip with eight pairs of floating electrodes and the frequency, voltage intensity, inlet flow and liquid conductivity dependencies of the mixing performance.

**Figure 7:**
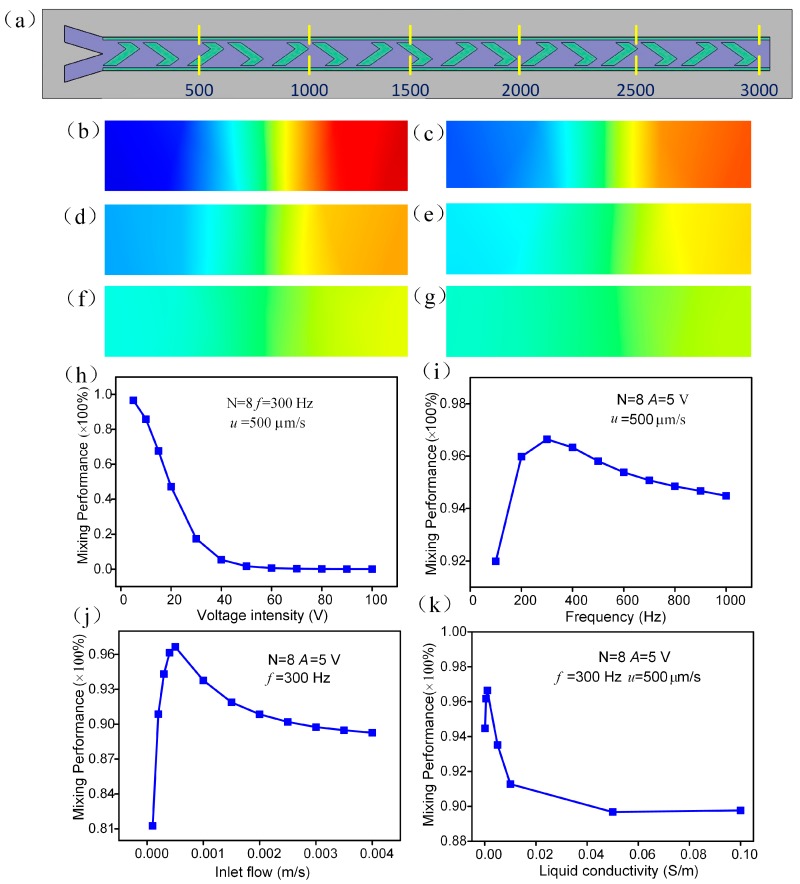
Parametric effect on mixing performance. (**a**–**g**) the concentration distribution at different cross section of channel at *A* = 5 V, *f* = 300 Hz, *u* = 500 μm/s. (**b**) The cross section at a range of 500 μm; (**c**) 1000 μm; (**d**) 1500 μm; (**e**) 2000 μm; (**f**) 2500 μm; (**g**) 3000 μm; (**h**) the voltage intensity effect on mixing performance at *f* = 300 Hz, *u* = 500 μm/s. (**i**) the frequency dependency of mixing performance at *A* = 5V, *u* = 500 μm/s; (**j**) the influence of inlet flow on mixing performance at *A* = 5 V, *f* = 300 Hz. (**k**) The relationship of mixing performance and liquid conductivity at *A* = 5 V, *f* = 300 Hz, *u* = 500 μm/s.

We defined six cross sections in the microchannel to investigate the sample concentration distribution under the ICEO action induced by different cycles of herringbone floating electrodes (Figure 7a). Figure 7b–g describes the fluid sample concentration distribution at different cross sections with the distance 500, 1000, 1500, 2000, 2500, and 3000 μm to the inlet at *A* = 5 V, *f* = 300 Hz, *u* = 500 μm/s. After the mixing effect of a cycle, only the fluid sample near the original interface is disturbed. At the distance of 1000 um to the inlet, the fluid sample near the left and right driving electrodes is transferred from the side walls to the middle and have certain mixing performance. After five cycles of herringbone floating electrodes, the concentration of the sample tends to be uniform. With the distance from the inlet increasing, the concentration reach a stable constant value, around 0.5. Figure 7h illustrates the relationship between the mixing performance and electrical field intensity at *f* = 300 Hz and *u* = 500 μm/s for eight pairs of herringbone floating electrodes. The mixing efficiency reaches up to 0.98 at *A* = 5 V, while the mixing efficiency diminishes with the increasing voltage amplitude. As mentioned above, the mixing tends to be saturated as the electrical field strength goes beyond a certain threshold, in which the resulting axial ICEO slip velocity component is big enough to confront the inlet flow velocity and even destroy the micromixer due to the large pressure. Therefore, the mixing efficiency is almost zero when the voltage amplitude is beyond 50 V under the same conditions for the eight pairs of herringbone floating electrodes. In particular, when the voltage intensity is over 50 V the mixing percentage tends to zero. The frequency dependency of the mixing efficiency is given in Figure 7i at *A* = 5 V, *u* = 500 μm/s. Noticeably, the mixing efficiency reach peak value 0.965 at around the RC relaxation frequency 300 Hz. The influence of inlet flow on the mixing behavior is shown in the Figure 7j, when *A* = 5 V, *f* = 300 Hz. The mixing percentage reaches peak value of 0.98 at the flow rate of 500 μm/s. The effect of the liquid conductivity on the mixing performance is given in Figure 7k at *A* = 5 V, *f* = 300 Hz, *u* = 500 μm/s, revealing that the device can achieve great mixing performance at the conductivity of 0.001 S/m. With the liquid conductivity increasing from 0.001 S/m, the mixing efficiency evidently declines.

## 4. Conclusions

In summary, we presented the asymmetrical ICEO flow on a herringbone floating electrode and developed a novel micromixer. The average transverse ICEO slip velocities on three proposed forms of floating electrodes were investigated and we found that the herringbone floating electrode structure has a good potential for mixing the fluidic samples in the microchannel. We then explored the cross flow on the herringbone floating electrode and analyzed the principle of disturbing the interface and delivering the fluid. Next, we investigated the influence of the number of herringbone floating electrodes on the mixing performance. Finally, we studied the dependencies of the micromixer mixing performance on voltage input and fluidic condition to characterize this device. The proposed micromixer with a herringbone floating electrode sequence can improve the mixing performance to some extent and provide great opportunities for efficient electrokinetic mixing with broad application in microfluidic systems.

## Figures and Tables

**Table 1:** The specific geometric size of the micromixer.

Parameter	W1	W2	W3	W4	W5	W6	*θ*
Value (μm)	50	40	150	30	200	100	90^o^

## References

[1] Kockmann N., Kiefer T., Engler M., Woias P. (2006). Convective mixing and chemical reactions in microchannels with high flow rates. Sens. Actuators B.

[2] Mariotti A., Galletti C., Mauri R., Salvetti M.V., Brunazzi E. (2018). Steady and unsteady regimes in a t-shaped micro-mixer: Synergic experimental and numerical investigation. Chem. Eng. J..

[3] Kim J., Jang Y., Byun D., Kim D.H., Kim M.J. (2013). Mixing enhancement by biologically inspired convection in a micro-chamber using alternating current galvanotactic control of the tetrahymena pyriformis. Appl. Phys. Lett..

[4] Pham N., Radajewski D., Round A., Brennich M., Pernot P., Biscans B., Bonnete F., Teychene S. (2017). Coupling high throughput microfluidics and small-angle x-ray scattering to study protein crystallization from solution. Anal. Chem..

[5] Jeong G.S., Chung S., Kim C.B., Lee S.H. (2010). Applications of micromixing technology. Analyst.

[6] Hansen C.L., Classen S., Berger J.M., Quake S.R. (2006). A microfluidic device for kinetic optimization of protein crystallization and in situ structure determination. J. Am. Chem. Soc..

[7] Duncombe T.A., Tentori A.M., Herr A.E. (2015). Microfluidics: Reframing biological enquiry. Nat. Rev. Mol. Cell Biol..

[8] Sackmann E.K., Fulton A.L., Beebe D.J. (2014). The present and future role of microfluidics in biomedical research. Nature.

[9] Hu Q., Ren Y., Liu W., Tao Y., Jiang H. (2017). Simulation analysis of improving microfluidic heterogeneous immunoassay using induced charge electroosmosis on a floating gate. Micromachines.

[10] Sharma S., Zapatero-Rodríguez J., Estrela P., O’Kennedy R. (2015). Point-of-care diagnostics in low resource settings: Present status and future role of microfluidics. Biosensors.

[11] Shen H.-H., Fan S.-K., Kim C.-J., Yao D.-J. (2014). Ewod microfluidic systems for biomedical applications. Microfluid. Nanofluid..

[12] Zhao Z., Yang Y., Zeng Y., He M. (2016). A microfluidic exosearch chip for multiplexed exosome detection towards blood-based ovarian cancer diagnosis. Lab Chip.

[13] Hessel V., Löwe H., Schönfeld F. (2005). Micromixers—A review on passive and active mixing principles. Chem. Eng. Sci..

[14] Lee C.-Y., Chang C.-L., Wang Y.-N., Fu L.-M. (2011). Microfluidic mixing: A review. Int. J. Mol. Sci..

[15] Cai G., Xue L., Zhang H., Lin J. (2017). A review on micromixers. Micromachines.

[16] Hong C.C., Choi J.W., Ahn C.H. (2004). A novel in-plane passive microfluidic mixer with modified tesla structures. Lab Chip.

[17] Hossain S., Ansari M.A., Husain A., Kim K.-Y. (2010). Analysis and optimization of a micromixer with a modified tesla structure. Chem. Eng. J..

[18] Lin Y., Gerfen G.J., Rousseau D.L., Yeh S.-R. (2003). Ultrafast microfluidic mixer and freeze-quenching device. Anal. Chem..

[19] Stroock A.D., Dertinger S.K.W., Ajdari A., Mezić I., Stone H.A., Whitesides G.M. (2002). Chaotic mixer for microchannels. Science.

[20] Marschewski J., Jung S., Ruch P., Prasad N., Mazzotti S., Michel B., Poulikakos D. (2015). Mixing with herringbone-inspired microstructures: Overcoming the diffusion limit in co-laminar microfluidic devices. Lab Chip.

[21] Leung A.K., Tam Y.Y., Chen S., Hafez I.M., Cullis P.R. (2015). Microfluidic mixing: A general method for encapsulating macromolecules in lipid nanoparticle systems. J. Phys. Chem. B.

[22] Ansari M.A., Kim K.-Y. (2009). Parametric study on mixing of two fluids in a three-dimensional serpentine microchannel. Chem. Eng. J..

[23] Liu R.H., Stremler M.A., Sharp K.V., Olsen M.G., Santiago J.G., Adrian R.J., Aref H., Beebe D.J. (2000). Passive mixing in a three-dimensional serpentine microchannel. J. Microelectromech. Syst..

[24] Feng X., Ren Y., Jiang H. (2014). Effect of the crossing-structure sequence on mixing performance within three-dimensional micromixers. Biomicrofluidics.

[25] Cannon D.M., Kuo T.-C., Bohn P.W., Sweedler J.V. (2003). Nanocapillary array interconnects for gated analyte injections and electrophoretic separations in multilayer microfluidic architectures. Anal. Chem..

[26] Wang Y., Zhe J., Chung B.T.F., Dutta P. (2008). A rapid magnetic particle driven micromixer. Microfluid. Nanofluid..

[27] Lee S.H., van Noort D., Lee J.Y., Zhang B.T., Park T.H. (2009). Effective mixing in a microfluidic chip using magnetic particles. Lab Chip.

[28] Wang S.S., Jiao Z.J., Huang X.Y., Yang C., Nguyen N.T. (2009). Acoustically induced bubbles in a microfluidic channel for mixing enhancement. Microfluid. Nanofluid..

[29] Ahmed D., Mao X., Juluri B.K., Huang T.J. (2009). A fast microfluidic mixer based on acoustically driven sidewall-trapped microbubbles. Microfluid. Nanofluid..

[30] Chen H., Gao Y., Petkovic K., Yan S., Best M., Du Y., Zhu Y. (2017). Reproducible bubble-induced acoustic microstreaming for bead disaggregation and immunoassay in microfluidics. Microfluid. Nanofluid..

[31] Huang C., Tsou C. (2014). The implementation of a thermal bubble actuated microfluidic chip with microvalve, micropump and micromixer. Sens. Actuators A.

[32] Jian Y. (2015). Transient mhd heat transfer and entropy generation in a microparallel channel combined with pressure and electroosmotic effects. Int. J. Heat. Mass Transfer.

[33] Li S.-X., Jian Y.-J., Xie Z.-Y., Liu Q.-S., Li F.-Q. (2015). Rotating electro-osmotic flow of third grade fluids between two microparallel plates. Colloids Surf. A.

[34] Zhao C., Yang C. (2018). Continuous-flow trapping and localized enrichment of micro- and nano-particles using induced-charge electrokinetics. Soft Matter.

[35] Prabhakaran R.A., Zhou Y., Zhao C., Hu G., Song Y., Wang J., Yang C., Xuan X. (2017). Induced charge effects on electrokinetic entry flow. Phys. Fluids.

[36] Hu Q., Ren Y., Liu W., Chen X., Tao Y., Jiang H. (2017). Fluid flow and mixing induced by ac continuous electrowetting of liquid metal droplet. Micromachines.

[37] Liu W., Wu Q., Ren Y., Cui P., Yao B., Li Y., Hui M., Jiang T., Bai L. (2018). On the bipolar dc flow field-effect-transistor for multifunctional sample handing in microfluidics: A theoretical analysis under the debye–huckel limit. Micromachines.

[38] Sasaki N., Kitamori T., Kim H.-B. (2006). Ac electroosmotic micromixer for chemical processing in a microchannel. Lab Chip.

[39] Melvin E.M., Moore B.R., Gilchrist K.H., Grego S., Velev O.D. (2011). On-chip collection of particles and cells by ac electroosmotic pumping and dielectrophoresis using asymmetric microelectrodes. Biomicrofluidics.

[40] Mirzajani H., Cheng C., Wu J., Ivanoff C.S., Najafi Aghdam E., Badri Ghavifekr H. (2016). Design and characterization of a passive, disposable wireless ac-electroosmotic lab-on-a-film for particle and fluid manipulation. Sens. Actuators B.

[41] Zhou T., Wang H., Shi L., Liu Z., Joo S. (2016). An enhanced electroosmotic micromixer with an efficient asymmetric lateral structure. Micromachines.

[42] Hughes M.P. (2016). Fifty years of dielectrophoretic cell separation technology. Biomicrofluidics.

[43] Song H., Rosano J.M., Wang Y., Garson C.J., Prabhakarpandian B., Pant K., Klarmann G.J., Perantoni A., Alvarez L.M., Lai E. (2015). Continuous-flow sorting of stem cells and differentiation products based on dielectrophoresis. Lab Chip.

[44] Liu W., Ren Y., Tao Y., Chen X., Wu Q. (2017). Electrode cooling effect on out-of-phase electrothermal streaming in rotating electric fields. Micromachines.

[45] Salari A., Navi M., Dalton C. (2015). A novel alternating current multiple array electrothermal micropump for lab-on-a-chip applications. Biomicrofluidics.

[46] Sigurdson M., Wang D., Meinhart C.D. (2005). Electrothermal stirring for heterogeneous immunoassays. Lab Chip.

[47] Tao Y., Ren Y., Liu W., Wu Y., Jia Y., Lang Q., Jiang H. (2016). Enhanced particle trapping performance of induced charge electroosmosis. Electrophoresis.

[48] Ren Y., Liu J., Liu W., Lang Q., Tao Y., Hu Q., Hou L., Jiang H. (2016). Scaled particle focusing in a microfluidic device with asymmetric electrodes utilizing induced-charge electroosmosis. Lab Chip.

[49] Yalcin S.E., Sharma A., Qian S., Joo S.W., Baysal O. (2010). Manipulating particles in microfluidics by floating electrodes. Electrophoresis.

[50] Paustian J.S., Pascall A.J., Wilson N.M., Squires T.M. (2014). Induced charge electroosmosis micropumps using arrays of janus micropillars. Lab Chip.

[51] Hossan M.R., Diganta D., Nazmul I., Prashanta D. (2018). Review: Electric field driven pumping in microfluidic device. Electrophoresis.

[52] Wu Y., Ren Y., Tao Y., Hou L., Jiang H. (2016). Large-scale single particle and cell trapping based on rotating electric field induced-charge electroosmosis. Anal. Chem..

[53] Zehavi M., Yossifon G. (2014). Particle dynamics and rapid trapping in electro-osmotic flow around a sharp microchannel corner. Phys. Fluids.

[54] Harrison H., Lu X., Patel S., Thomas C., Todd A., Johnson M., Raval Y., Tzeng T.-R., Song Y., Wang J. (2015). Electrokinetic preconcentration of particles and cells in microfluidic reservoirs. Analyst.

[55] SQUIRES T.M., BAZANT M.Z. (2004). Induced-charge electro-osmosis. J. Fluid Mech..

[56] Ren Y., Liu W., Jia Y., Tao Y., Shao J., Ding Y., Jiang H. (2015). Induced-charge electroosmotic trapping of particles. Lab Chip.

[57] Wu Y., Ren Y., Tao Y., Hou L., Hu Q., Jiang H. (2017). A novel micromixer based on the alternating current-flow field effect transistor. Lab Chip.

[58] Ren Y., Liu W., Tao Y., Hui M., Wu Q. (2018). On ac-field-induced nonlinear electroosmosis next to the sharp corner-field-singularity of leaky dielectric blocks and its application in on-chip micro-mixing. Micromachines.

[59] Chen X., Ren Y., Liu W., Feng X., Jia Y., Tao Y., Jiang H. (2017). A simplified microfluidic device for particle separation with two consecutive steps: Induced charge electro-osmotic prefocusing and dielectrophoretic separation. Anal. Chem..

[60] Bazant M.Z., Squires T.M. (2010). Induced-charge electrokinetic phenomena. Curr. Opin. Colloid Interface Sci..

[61] Lin Y., Yu X., Wang Z., Tu S.-T., Wang Z. (2011). Design and evaluation of an easily fabricated micromixer with three-dimensional periodic perturbation. Chem. Eng. J..

[62] Xuan X., Li D. (2005). Electroosmotic flow in microchannels with arbitrary geometry and arbitrary distribution of wall charge. J. Colloid Interface Sci..

[63] Bazant M.Z., Kilic M.S., Storey B.D., Ajdari A. (2009). Towards an understanding of induced-charge electrokinetics at large applied voltages in concentrated solutions. Adv. Colloid Interface.

[64] Xuan X., Xu B., Sinton D., Li D. (2004). Electroosmotic flow with joule heating effects. Lab Chip.

[65] Hu G., Li D. (2007). Multiscale phenomena in microfluidics and nanofluidics. Chem. Eng. Sci..

